# Management of a Penile Fracture With Concurrent Urethral Injury: A Case Report

**DOI:** 10.7759/cureus.75751

**Published:** 2024-12-15

**Authors:** Turan Ozdemir, Akif Erbin, Enes Aktas, Cemal Topal, Halil L Canat

**Affiliations:** 1 Department of Urology, Basaksehir Cam Sakura City Hospital, Istanbul, TUR

**Keywords:** penile diseases, penile fracture, sexual intercourse, urethral injury, urological emergency

## Abstract

A penile fracture is typically a urological emergency resulting from blunt trauma to the penis, particularly during sexual activity, and it is rarely associated with urethral injury. A 52-year-old male patient presented to our emergency department with complaints of penile swelling and bruising following sexual intercourse. Assessment of the patient indicated the presence of both penile fracture and anterior urethral injury, and simultaneous repairs were performed. End-to-end anastomosis urethroplasty was conducted utilizing a 4/0 polyglactin suture in a separate manner. The penile fracture line was subsequently repaired using a 3/0 polyglactin suture in a watertight manner, employing a separate suture technique. This case report study provides a detailed discussion of the patient's clinical management and treatment process, as well as a thorough examination of the existing current literature.

## Introduction

Penile fracture (PF) is a rare urological emergency, and it typically occurs when a rigid penis strikes the partner's perineum or pubic bone during sexual intercourse [1]. A recent meta-analysis has indicated that the doggy style and man-on-top positions pose the highest risk for PF during intercourse [2]. Aside from sexual intercourse, masturbation injuries, falls on an erect penis, rolling over during sleep, and forcefully bending the erect penis downwards to cause rapid detumescence (called taqaandan) are less common etiologies of penile fracture [3].

Penile fracture associated with urethral injury is classified as rare, occurring at a rate of 3.9% [4]; however, it is a substantial urological emergency. Failure to manage this condition appropriately may result in severe complications such as erectile dysfunction, urethral stricture, urinary retention, urinary fistula formation, penile curvature or deformity, infection or abscess formation, loss of penile length, and avascular necrosis. In this study, we present a case of PF with urethral involvement and provide a comprehensive discussion of its clinical presentation, management, and outcomes with the current literature.

## Case presentation

**Figure 1:**
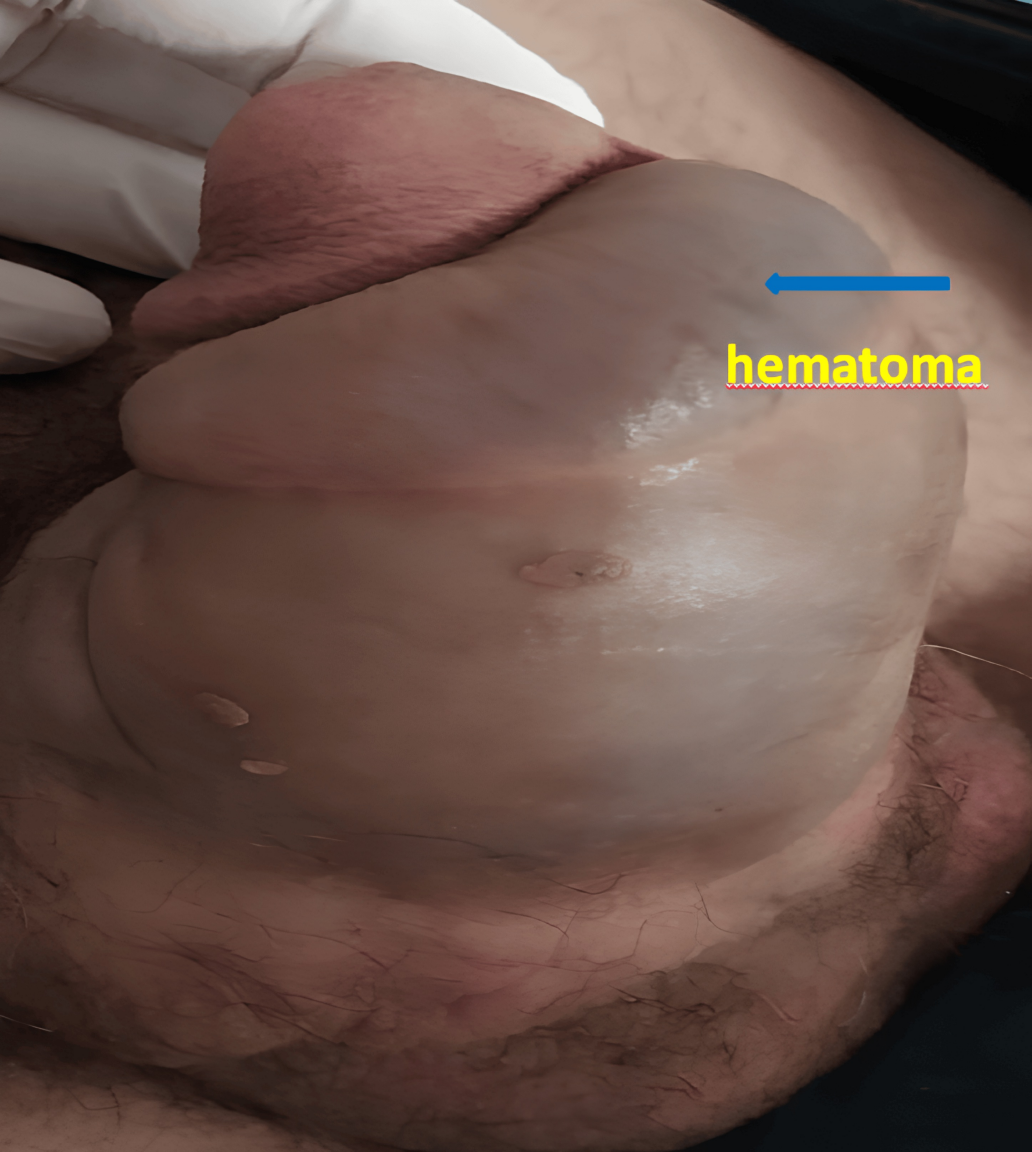
“Eggplant” deformity sign (blue arrow)

The required consent was obtained from the patient for the publication of this case report study. A 52-year-old male patient presented to our emergency department with complaints of penile swelling and bruising. He arrived approximately one-hour post-trauma during sexual intercourse. The patient's history indicated a sudden noise, pain, and an abrupt loss of erection during sexual intercourse. The physical examination revealed a characteristic 'eggplant' deformity of the penis (Figure 1) and the presence of blood at the urethral meatus. The patient had no history of systemic disease or prior medication. There was no past surgical history for the patient. Penile surface tissue ultrasound revealed a hyper-echoic area consistent with a hematoma measuring up to 4 cm at its thickest point. A hypoechoic area was observed in the middle segment of the tunica albuginea of the penis, indicating a potential discontinuity. Following the clinical evaluation, physical examination, and imaging results, the patient underwent surgical intervention for suspected penile fracture and urethral injury.

**Figure 2:**
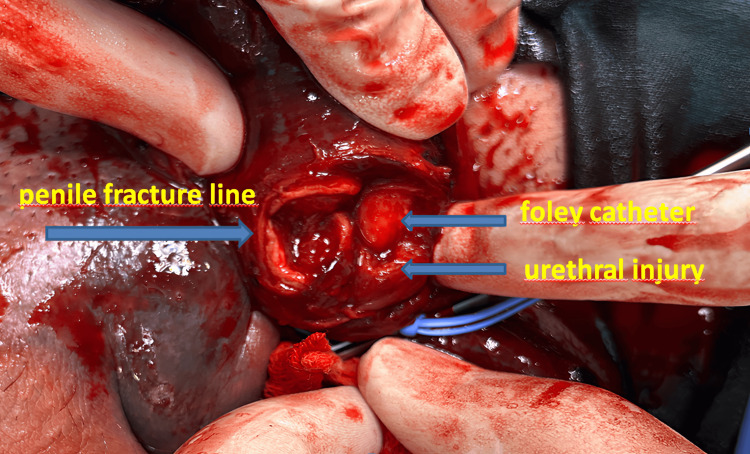
Operative image of corpus cavernosum rupture, urethral injury, and urethral catheter (blue arrows)

Following the administration of spinal anesthesia and cleaning of the surgical area, circumferential incision and penile degloving were performed. A hematoma measuring 3x3 cm was identified beneath Buck's fascia on the right ventrolateral side of the penile shaft, near the penile root, at the one-third level. A vertical incision was performed over the hematoma, followed by evacuation and cleansing of the site. A 2 cm fracture line measuring approximately 250-270 degrees in extension was identified in the right corpus cavernosum at the one-third level, ventrolaterally. Additionally, an anterior urethral injury was identified adjacent to the fracture line at the same level, with approximately 270 degrees of urethral separation observed at the location of the injury (Figure 2). Following the careful placement of a 16 Fr Foley catheter, the urethral ends were easily approximated and subsequently joined with a spatula to achieve an end-to-end anastomosis urethroplasty. The urethral repair was conducted utilizing a 4/0 polyglactin suture (®Medsorb PGLA, Medeks Inc., Istanbul, Turkey) in a separate manner. The penile fracture line was subsequently repaired using a 3/0 polyglactin suture in a watertight manner, employing a separate suture technique. Subsequently, an artificial erection was induced using a butterfly needle and saline, and the suture line was examined for leakage; no leakage was observed. Following the control of hemostasis, the layers were closed anatomically, and the procedure was completed. The wound was dressed with a Coban bandage.

On the second postoperative day, the patient was discharged with a catheter in situ. Wound care instructions were given, and the patient was advised to refrain from sexual intercourse for a duration of six weeks. The Foley catheter was removed two weeks later. At the one-month and three-month follow-up visits, the patient reported no urinary issues. An assessment of erectile function (ED) revealed no significant difference prior to the penile fracture and following the surgical intervention. The patient expressed satisfaction with the functional outcome and cosmetic results.

## Discussion

Urethral injury associated with PF occurs infrequently; however, the incidence of PF and the likelihood of concurrent urethral injury differ across various populations. The probability of concurrent urethral injury is greater in European countries than in Asian countries. However, reports indicate that a low proportion of penile fractures in the Middle East and North Africa are associated with the prevalent practice of "taqaandan (to click or snap while applying force to depress the erect penis for the purpose of achieving detumescence)," which involves low-energy trauma and presents a minimal risk of urethral injuries [5]. The impact of urethral rupture on functional outcomes, particularly ED, remains a subject of debate. A recent study in the Turkish population indicated that urethral rupture repair did not negatively impact functional outcomes or elevate penile complication rates [6]. Conversely, a study in the Chinese population found a significant correlation between urethral injury associated with penile fracture and postoperative ED [7]. The primary factors influencing erectile dysfunction in PF, regardless of urethral injury, include the adequacy of surgical repair and the timing of the surgical intervention. A recent review indicated that immediate surgical repair (within 24 hours post-injury) yields superior outcomes and that surgical intervention should be contemplated even in instances of delayed presentation (beyond 24 hours post-injury) [8]. We attribute the absence of early ED in our patient to the timely and appropriate intervention as well as the absence of any comorbidities in the patient.

While the probability of urethral injury is elevated in fractures of the proximal penile regions, it can also manifest with distal PFs observed at an atypical site between the corpora cavernosa and the corpora spongiosum laterally [9]. So it must be kept in mind that urethral damage can occur in conjunction with all PFs. In the event that a urethral rupture is missed, it could lead to a number of problems, including the formation of an abscess, penile curvature, extravasation, fibrosis, plaque, hematuria, nodules at the injury site, painful erections, painful intercourse, urethral stricture, urinary retention, urinoma formation, and a weak urinary stream. A recent review of 1,671 patients with penile fractures revealed that 65 patients had concurrent urethral injuries. At a median follow-up of 21 months (range, 1-107 months) post-primary urethroplasty and penile fracture repair, five patients (8.5%) developed urethral stricture. Other reported complications included penile curvature (6.7%), palpable fibrosis (6.7%), and erectile dysfunction (3.4%) [4].

## Conclusions

The level of suspicion regarding urethral injury in a PF should be high. While the impact of urethral injury on ED remains a topic of debate, it is essential not to disregard the potential complications associated with urethral injury that may occur alongside PF, and as soon as possible, it should have the proper surgical treatment. Delayed action or inadequate surgical intervention may result in consequences that are challenging to manage.
